# Highlight report: Blueprint for stem cell differentiation into liver cells

**DOI:** 10.17179/excli2015-549

**Published:** 2015-09-02

**Authors:** Ahmed Ghallab

**Affiliations:** 1 Forensic Medicine and Toxicology Department, Faculty of Veterinary Medicine, South Valley University, Qena, Egypt

## ‬‬‬

Stem cell research is of high interest, because precursor cells can be directed to differentiate into practically all mature cell types. This represents a potential perspective for therapeutics of severely damaged organs and also an alternative to animal drug and toxicity testing. Nonetheless, an essential question still remains: How similar are differentiated stem cells to the desired mature cells, e.g. liver cells? This highlight report focusses on a study recently published in the Journal of Hepatology (Godoy et al., 2013[10]) where three European research centers developed a method for precisely assessing the degree of stem cell-differentiation into hepatocytes based on whole-genome gene expression analysis and statistical models.

The liver has a spectacular ability to regenerate (Hoehme et al., 2010[16]; Drasdo et al., 2014[2]; Schliess et al. 2014[19]; Nussler et al., 2014[18]). However, this capacity is compromised after severe acute damage or in chronic disease such as cirrhosis. In these situations, the only solution up to now is organ transplantation, which comes often too late and implies high risks for the patients. Scientists around the world are working on a promising alternative: stem cell therapy (Brulport et al., 2007[1]). In principle, stem cells have the capacity to differentiate into every cell of the human body - skin, neuronal cells or hepatocytes. However, the degree of similarity between stem cell-derived tissue cells (for example, hepatocyte-like cells) and primary hepatocytes is still controversial (Godoy et al., 2013[10]; Hengstler et al., 2005[14]). This is a very important issue, because the application of these cells for therapeutic or toxicity testing requires that they perform all functions of a mature tissue.

Recently Godoy et al. (2015[11]) developed a method for comparing hepatocyte-like cells and original (primary) human hepatocytes based on their gene expression profiles. Given the high number of genes, approximately 22.000, in the human genome, this comparison is not an easy task. The authors clustered the thousands of genes according to special functions and regulatory principles by applying mathematical models. For example, some genes are responsible for the expression of proteins involved in metabolism, while others regulate cell proliferation. Particularly relevant for hepatocytes are cytochrome P450 enzymes and phase II metabolizing enzymes, because they decompose toxic substances - a main task of the liver. On the other hand, genes regulating the cell cycle are less relevant because hepatocytes do not proliferate at a high rate in a healthy liver (Zellmer et al., 2010[22]). 

The authors applied biostatistical techniques to compare gene clusters using real liver cells (primary human hepatocytes), stem cells and six different stem cell-derived hepatocyte-like cells (Godoy et al., 2015[11]). The comparison shows that in some gene clusters hepatocyte-like cells are very similar to primary hepatocytes, including many genes involved in drug and xenobiotic metabolism. Surprisingly, other gene clusters in HLC indicate that these cells acquire properties of additional tissues, including colon and fibroblasts. Importantly, the analysis revealed the mechanisms and transcription factors responsible for the expression of genes associated with each tissue type. This approach allows to precisely determine the extent of differentiation from stem cells, the degree of acquisition of liver features and the appearance of undesired additional tissue types.

The study of Godoy et al. (2015[11]) represents an important step towards a more precise and unbiased determination, to which degree stem cell derived cells resemble primary cells. Furthermore, since cultivated liver cells are a basic tool for testing the effects of new medicinal drugs, hepatocyte-like cells can become an important alternative to animal testing (Ghallab et al., 2013[5], 2014[4][6]). Currently such alternative *in vitro* systems play a major role in testing of neurotoxicity (Waldmann et al., 2014[20]; Zimmer et al., 2014[23]; Krug et al., 2013[17]), nephrotoxicity (Faiz et al., 2015[3]; Yang et al., 2014[21]) and hepatotoxicity (Heise et al., 2012[13]; Godoy et al., 2009[9]; Godoy 2011[7]; Godoy and Bolt, 2012[8]; Grinberg et al., 2014[12]; Hengstler et al., 2000[15]). The results are also important, because medicinal and toxic substances are currently being tested with hepatocyte-like cells, and now it becomes possible to determine how trustworthy the results can be.

This is the first time that such a systematic genome wide comparison of stem cell derived and genuine hepatocytes has been performed. The recent published study in the Journal of Hepatology (Godoy et al., 2015[11]) offers a blueprint for research in stem cell differentiation of liver cells.
